# Brain atrophy measurement over a MRI scanner change in multiple sclerosis

**DOI:** 10.1016/j.nicl.2022.103148

**Published:** 2022-08-10

**Authors:** Tim Sinnecker, Sabine Schädelin, Pascal Benkert, Esther Ruberte, Michael Amann, Johanna M. Lieb, Yvonne Naegelin, Jannis Müller, Jens Kuhle, Tobias Derfuss, Ludwig Kappos, Jens Wuerfel, Cristina Granziera, Özgür Yaldizli

**Affiliations:** a Neurologic Clinic and Policlinic, Departments of Head, Spine and Neuromedicine, Clinical Research and Biomedical Engineering, University Hospital Basel and University of Basel, Basel, Switzerland; b Translational Imaging in Neurology [ThINK] Basel, Departments of Head, Spine and Neuromedicine and Biomedical Engineering, University Hospital Basel and University of Basel, Basel, Switzerland; c Medical Image Analysis Center (MIAC AG) and Department of Biomedical Engineering, University of Basel, Basel, Switzerland; d Department of Clinical Research, Clinical Trial Unit, University Hospital Basel and University of Basel, Basel, Switzerland; e Department of Neuroradiology, University Hospital Basel and University of Basel, Basel, Switzerland; f Research Center for Clinical Neuroimmunology and Neuroscience (RC2NB), University Hospital and University of Basel, Switzerland

**Keywords:** MRI Scanner change, Multiple sclerosis, Brain atrophy, Cohort study

## Abstract

**Background:**

A change in MRI hardware impacts brain volume measurements. The aim of this study was to use MRI data from multiple sclerosis (MS) patients and healthy control subjects (HCs) to statistically model how to adjust brain atrophy measures in MS patients after a major scanner upgrade.

**Methods:**

We scanned 20 MS patients and 26 HCs before and three months after a major scanner upgrade (1.5 T Siemens Healthineers Magnetom Avanto to 3 T Siemens Healthineers Skyra Fit). The patient group also underwent standardized serial MRIs before and after the scanner change. Percentage whole brain volume changes (PBVC) measured by Structural Image Evaluation using Normalization of Atrophy (SIENA) in the HCs was used to estimate a corrective term based on a linear model. The factor was internally validated in HCs, and then applied to the MS group.

**Results:**

Mean PBVC during the scanner change was higher in MS than HCs (-4.1 ± 0.8 % versus −3.4 ± 0.6 %). A fixed corrective term of 3.4 (95% confidence interval: 3.13–3.67)% was estimated based on the observed average changes in HCs. Age and gender did not have a significant influence on this corrective term. After adjustment, a linear mixed effects model showed that the brain atrophy measures in MS during the scanner upgrade were not anymore associated with the scanner type (old vs new scanner; p = 0.29).

**Conclusion:**

A scanner change affects brain atrophy measures in longitudinal cohorts. The inclusion of a corrective term based on changes observed in HCs helps to adjust for the known and unknown factors associated with a scanner upgrade on a group level.

## 1 Introduction

Multiple sclerosis (MS) is traditionally seen as an inflammatory disease of autoimmune origin. (Filippi et al., 2018, Longo et al., 2018) However, in the last decades it became clear that neurodegeneration is present together with inflammation even in the earliest stages of the disease. (Filippi et al., 2018, Longo et al., 2018, Uher et al., 2021) As brain atrophy reflects neurodegeneration, brain volume changes detected by magnetic resonance imaging (MRI) have emerged as an important outcome measure in clinical trials. (Sastre-Garriga et al., 2020) Several disease modifying drugs have shown effects on brain volume change in MS (Sormani et al., 2014, Branger et al., 2016, Tsivgoulis et al., 2015). However, the measurement of brain volume change is challenging. Indeed, both biological (e.g. hydration status, inflammation, age, smoking, alcohol or diet) and technical factors affect MRI-based brain volume measurements. (Sinnecker et al., 2018) Scanner dependent factors such as magnetic field strength, gradient system, coils as well as MRI pulse sequence parameters largely influence the MR image quality and the performance of software assessing brain volume changes over time. Tissue contrast, signal-to-noise ratio, signal homogeneity and geometric distortions affect the segmentation of brain structures. (Sinnecker et al., 2018, Preboske et al., 2006) Thus, a change in scanner hardware significantly impacts longitudinal brain volume measurements, (Kruggel et al., 2010, Ghione et al., 2018) even when identical field strength, manufacturer and imaging protocol are used. (Shinohara et al., 2017).

Structural Image Evaluation using Normalization of Atrophy (SIENA) is one of the most commonly used software packages to assess whole brain volume changes in longitudinal studies. (Smith et al., 2002, Smith et al., 2004) SIENA corrects for differences in imaging geometry by using the outer skull surface as a reference and has been proposed to minimize the technical bias when analyzing brain volume changes. (Smith et al., 2002, Smith et al., 2004).

Yet, SIENA cannot intrinsically overcome the other technical factors associated with a scanner change mentioned above.

The aim of this study was to test whether the inclusion of data from healthy control subjects (HCs) scanned before and after a major scanner upgrade can be used to statistically model and adjust for the effect of the scanner change on brain atrophy measurements in MS patients in a real-world setting.

## 2 Methods

In 2016, the MRI scanner at the University Hospital Basel was upgraded from 1.5 Tesla to 3.0 Tesla (see more details below). Based on the reported measurements and variability of annual PBVC in MS and HCs in a representative cohort, (De Stefano et al., 2016) sample size calculations revealed a number n = 20 HCs to be sensitive to changes in PBVC of 0.24 %. Sample size calculations were based on a two-sample *t*-test (significance level 0.05, power 0.8) with the assumption that variability in PBVC does not change over the scanner change. Thus, we scanned 20 MS patients from the Swiss MS cohort study and 26 HCs before and after the scanner change (median time interval 3.5 months, range 1.7–5.2 months). In the patient group, 6–14 MRIs per patient before (median follow up 9.8 years) and 2–3 MRIs after (median follow up 24.4 months) the scanner change were also available.

### 2.1 Clinical assessement

We used the expanded disability status scale applied by certified raters to assess disability in MS.

### 2.2 Ethics approval

The study was performed in accordance with the 1964 Declaration of Helsinki and its later amendments. It was approved by the ethics committee of North West and Central Switzerland. All participants signed an informed consent.

### 2.3 MRI acquisition

Brain MRIs were performed before and after a scanner change (1.5 T Magnetom Avanto to 3 T Skyra Fit; both Siemens Healthineers, Germany). A manufacturer-supplied 12-channel (1.5 T) or 20-channel (3.0 T) phased-array head coil was used for reception. For transmission, the built-in body coil was employed. The imaging protocol included a T1-weighted MPRAGE at 1.5 T [echo time (TE) = 3 ms, repetition time (TR) = 2080 ms, inversion time (TI) = 1100 ms, spatial resolution: 1.0 × 0.977 × 0.977 mm^3^], and 3.0 T [TE = 3 ms, TR = 2300 ms, TI = 900 ms, spatial resolution: 1.0 × 1.0 × 1.0 mm^3^], and a two-dimensional axial multi-echo sequence with proton density and T2 weighted (T2w) contrast [spatial resolution: 3.0 × 0.977 × 0.977 mm^3^]. All scans were corrected for B1 inhomogeneities and for gradient distortions by the scanners’ built-in procedures.

### 2.4 MRI analysis

SIENA as part of FMRIB Software Library (FSL, version 5.0.10, FMRIB Analysis Group, Oxford, UK) was used to measure percentage whole brain volume change (PBVC) on T1-weighted (T1w) MPRAGE with optimal parameters for brain extraction (bias field and neck cleanup option “B”, and fractional intensity threshold “f” of 0.25). (Smith et al., 2002, Smith et al., 2004) All SIENA outputs were reviewed for quality by ER and TS. None of the segmentations were excluded.

T2w lesions were manually marked by trained raters in consensus reading using JIM (Xinapse Systems ltd, West Bergholt, UK, see also supplemental material for more details).

### 2.5 Statistics

Mean PBVC of HCs was used to determine a corrective factor to adjust for BVC in MS patients attributed to the scanner change. In addition, a gender- and age-dependent factor was determined based on a linear model. Both factors were internally validated in the HCs using leave-one-out jackknife validation. In a second step, both correction factors were applied to the PBVC of MS patients and the raw as well as the corrected data was modeled in a mixed effects model, including the time point as fixed and patient as random effect. In MS patients, the variance in PBVC before and after the scanner change was assessed using an F-test of equality of variances. Normality of PBVC was assessed using quantile–quantile plots (QQ-plots). Model assumptions were controlled via inspection of residuals. The significance level was set to p < 0.05.

To test whether the association between EDSS and the brain volume change differ before vs after the scanner change with and without the correction term, we used a linear mixed model with EDSS at the end of each year as dependent variable. Furthermore, the model was adjusted for EDSS at the beginning of each year. We also included a binary variable that indicates whether the data was measured before or after the scanner change and tested the interaction between the binary variable and percentage brain volume change. A significant interaction would indicate that the association between EDSS and brain volume change differs, depending on whether brain volume change is measured using the old or the new scanner. The patient identifier was included as a random factor. Brain volume change measures in the year of the scanner change were excluded from the model. To test whether the correction of the brain volume change using data from the healthy individuals improves the association between brain volume change and EDSS, we fitted a new linear mixed model. We used the same model as the first one, but this time without the binary variable (before scanner change vs after scanner change) and the interaction term. Moreover, we included brain volume change over the scanner change (first MRI on old scanner, second MRI on new scanner) in this model.

Statistical analyses were performed by SS in the Clinical Trial Unit, University Hospital Basel using R (www.r-project.org).

## 3 Results

**Fig. 1:**
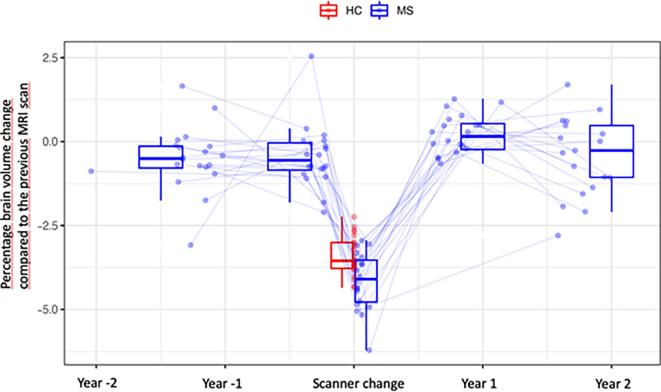
**Unadjusted brain atrophy measures in MS patients and healthy control subjects before, during and after the scanner change.** Abbreviations: HCs healthy control subjects; MS multiple sclerosis.

**Fig. 2:**
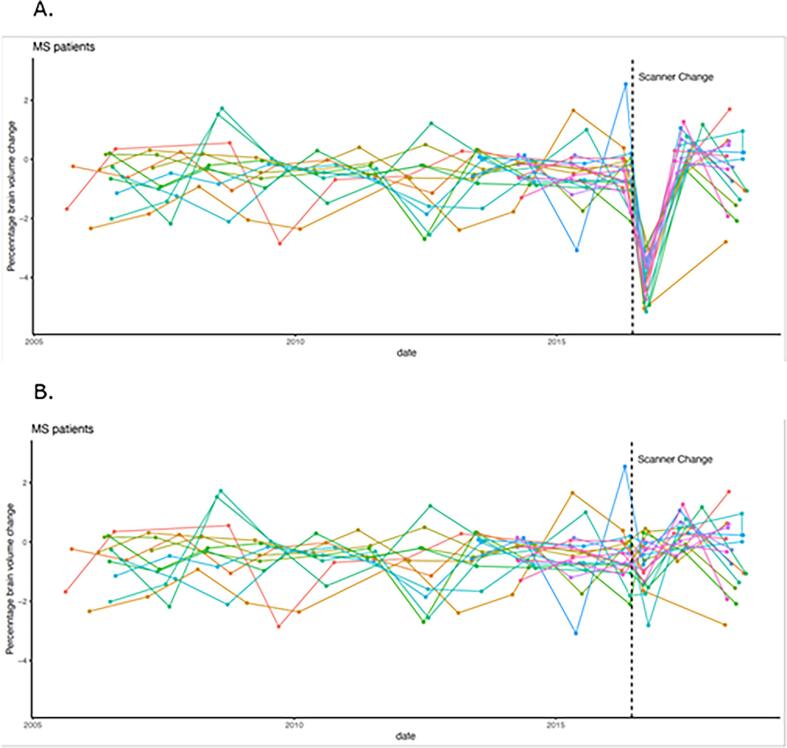
Unadjusted (A) brain atrophy measures in multiple sclerosis patients before, during and after the scanner upgrade in comparison to adjusted (B) brain atrophy measures in multiple sclerosis patients during the scanner upgrade. Statistical adjustment was based on changes observed in healthy controls.

Demographical and clinical details are presented in Table 1. During the scanner change, the mean PBVC was higher in MS than HCs (-4.1 ± 0.8 % vs −3.4 ± 0.6 %; estimate of the difference between MS and HCs 0.74, CI = [0.28,1.20], p-value: 0.0025, Fig. 1, 2). In MS, the variance in PBVC before and after the scanner change was similar (F-test, ratio = 1.15, 95 % CI = 0.46–2.91, p = 0.78).

**Table 1:** Demographic and clinical details.

	**Multiple sclerosis**	**Healthy controls**
**N**	20	26
**Age, years; mean ± SD [range]**	55 ± 15 [35–76]	31 ± 8 [24–71]
**Female, n [%]**	15 [75 %]	12 [46 %]
**Relapsing-remitting MS**	14 (70 %)	n/a
**Secondary progressive MS**	6 (30 %)*	n/a
**Disease duration, years; mean ± SD**	25.3 ± 14.5	n/a
**EDSS; median [range]**	3.0 [0–8]	n/a
**T2w lesion volume, cm ^3^;** mean **± SD [range]**	9.8 ± 8.3 [0.8 – 31.3]	0 ± 0.1 [0 – 0.2]

*one of the patients converted from RRMS to SPMS between the MRI scans 2015 and 2016 and this patients is counted here as SPMS.

Key: EDSS Expanded Disability Status Scale; 40% of the MS patients had progressive MS.

Bias field correction, histogram matching of signal intensities or lesion filling did not improve PBVC measurements over the scanner change (Supplemental Figs. 1 and 2).

### 3.1 Adjustment of brain volume measures during scanner change in HCs

A fixed corrective term of 3.4 % (95 % confidence interval: 3.13–3.67) was determined based on the observed average changes in HCs. Age and gender did not have a significant influence on this corrective term (data not shown). Internal validation provided a variance of 0.35 % for the jackknife estimator.

### 3.2 Adjustment of brain volume measures during scanner change in MS

In MS, the observed PBVC was adjusted by adding the fixed corrective term based on the observed changes in HCs. The adjusted PBVC during the scanner change was −0.7 ± 0.8 %. A linear mixed effects model confirmed this adjustment as the adjusted PBVCs during the scanner upgrade were not anymore associated with the scanner type (effect of the scanner change on PBVC before adjustment −3.6 ± 0.2 %, effect of the scanner change on PBVC after adjustment −0.2 ± 0.2 %; p = 0.29, Fig. 2B).

### 3.3 Effect of adjustment of brain volume measures during scanner change on a potential association with EDSS in MS

In our dataset we had 135 EDSS values from clinical visits that are associated with a MRI scan (+/- 120 days between MRI and EDSS assessment): 97 before and 38 after the scanner change. The median number of EDSS measurements per patient was 4 before the scanner change and 2 after the scanner change.

When excluding EDSS measures over the scanner change, we found, as expected, a strong association between EDSS at the end of the year and EDSS at the reference scan (Beta = 1.007; 95 %CI: [0.959,1.054]). Patients with brain atrophy had higher EDSS values. Each percentage brain volume loss was associated with an average increase of 0.14 points on the EDSS (Beta: −0.139; 95 %CI: [-0.345,0.068]). However, this effect was not statistically significant. No evidence for an interaction between type of MRI scanner (before scanner change vs after scanner change) and brain volume change could be found (p-value: 0.639).

When including the uncorrected EDSS measures over the scanner change, we found that there is still no significant association between EDSS and brain volume change (Beta = -0.033, 95 %CI = [-0.100,0.034], p = 0.343). However, when refitting the model using the adjusted brain volume change values, the estimates improved:

$$\text{korr}.\;\text{F1}:\;\text{Beta}=-0.0\text{92};\;\text{95}\%\text{CI}\;=\;\left[-0.\text{193},0.0\text{1}0\right];\;\text{p}=0.0\text{815}.$$

$$\text{korr}.\;\text{F2}:\;\text{Beta}=-0.0\text{92};\;\text{95}\%\;\text{CI}\;=\;\left[-0.\text{194},0.0\text{1}0\right];\;\text{p}=0.0\text{797}.$$

Again, the EDSS at the reference scan was highly correlated with EDSS after 1 year.

## 4 Discussion

The change in brain volume in longitudinal studies is an important measure of neurodegeneration in MS. (Sastre-Garriga et al., 2020, Sinnecker et al., 2018, Ghione et al., 2018) Unfortunately, scanner changes/upgrades are a common fact and usually unavoidable. In this study we used MRI data in HCs before and after a scanner change to adjust for the effect of the scanner change in MS patients.

Our study is in line with other studies (Kruggel et al., 2010, Ghione et al., 2018) showing that the effect of the scanner change was manifold higher than a potential disease-related or other biological effect on brain volumes. Moreover, we showed that the adjustment performed using brain volume changes from HCs during a scanner change reduces the effect of the scanner change on brain atrophy rates in the MS population.

Correcting for the effects of a scanner change is challenging as various biological and technical factors influence brain volume measurements which makes the estimation of true brain volume changes virtually impossible. Our study adds to previous work on linear, multivariable or mixed effect regression models used to overcome the heterogeneity of MRI data in longitudinal studies. (Jones et al., 2013, Lee et al., 2019) The use of data from a control group as correction factor has the potential advantage to also correct for unknown, but not disease related factors on brain volume changes.

A disadvantage of the statistical approach in general is that it requires reliable analysis beforehand to be applied when the scanner change occurs. Moreover, the corrective term represents an average on a group level and is not an adjustment on patient-level.

To better overcome the problem of MRI data inhomogeneity on the patient-level, techniques of MRI contrast harmonization before applying the specific MRI analysis software (e.g. SIENA) have been proposed: Some studies suggested to adjust image histograms (Shinohara et al., 2014) others tried to remove artificial voxel effects using linear regression analysis. (Fortin et al., 2016) Another recently proposed promising method to harmonize contrast between MR images is using deep learning-based techniques: (Armanious et al., 2020) Armanious et al. utilized a discriminator network as a trainable feature extractor to control for the discrepancy between the translated scan and the desired image modalities. Using this network, the authors were able to translate positron emission tomography scans into CT scans and correct for MRI motion artifacts. (Armanious et al., 2020) Dewey et al. were able to reduce inconsistencies in brain atrophy measurements across different MRI protocols by using a U-Net based deep learning architecture. (Dewey et al., 2019) These techniques seem to be promising but were not fully validated in MS yet. Also, they are probably more susceptible to the quality of the input images and the training dataset.

Our study is not without limitation: One limitation is that the old scanner was removed after the scanner change so that we could not scan the HCs in the old scanner parallel to the new scanner to assess brain atrophy in the HCs during the scanner change . We assumed that HCs had no systematic brain volume change during the time of scanner change but this is a biological simplification. Factors like hydration state, alcohol intake and time of scanning may have contributed to the variation observed. Nevertheless, our approach reflects a real-world scenario in observational cohort studies, and sample size calculation suggests that the approach is sensitive to changes in brain volume that differentiates brain atrophy rates in MS from HCs (De Stefano et al., 2016) if a sufficiently low variance can be achieved by reducing the variability caused by biological factors. The estimated corrective term is case-specific and cannot be generalized. Consequently, for a multicenter cohort, it is necessary to adjust this correction for each and every participating center that has a scanner change. The inclusion of a corrective term may help to adjust for the known and unknown factors associated with a scanner upgrade on a group level. Whether this adjustment improves the investigation of a potential association between brain volume changes (over a scanner change) and clinical outcome variables such as EDSS should be addressed in future studies with larger samples. In our analyses the adjustment of brain volume change over the scanner change improved the estimates, but these results come with the caveat of having a relatively small sample size to investigate the association between EDSS and brain atrophy properly.

## 5 Conclusion

A scanner change affects brain volume measurements in longitudinal cohorts. The inclusion of a corrective term based on changes observed in HCs adjusts for the variability associated with a scanner change on a group level.

## 6 Data sharing and data accessibility

Data may be shared for research purposes after qualified written request to the corresponding author.

## 7 Ethics approval

The study was performed in accordance with the 1964 Declaration of Helsinki and its later amendments. It was approved by the ethics committee of North West and Central Switzerland.

## 8 Consent to participate and consent for publication

All participants signed an informed consent.

## 9 Authors' contributions

Jens Kuhle, Tobias Derfuss, Ludwig Kappos, Jens Wuerfel, Cristina Granziera, and Özgür Yaldizli designed and conceptualized the study.

Tim Sinnecker, Sabine Schädelin, Esther Ruberte, Vera Canova, Michael Amann, and Özgür Yaldizli analysed the data.

Tim Sinnecker, Sabine Schädelin, Esther Ruberte, Michael Amann, Johanna M. Lieb, Yvonne Naegelin, Jannis Müller, Jens Kuhle, Tobias Derfuss, Ludwig Kappos, Jens Wuerfel, Cristina Granziera, and Özgür Yaldizli interpreted the data.

Tim Sinnecker, Jens Wuerfel, Cristina Granziera, and Özgür Yaldizli drafted the manuscript. All authors revised the manuscript.

## CRediT authorship contribution statement

**Tim Sinnecker:** Data curation, Formal analysis, Methodology, Software, Validation, Visualization, Writing – original draft. **Sabine Schädelin:** Data curation, Formal analysis, Methodology, Writing – review & editing. **Pascal Benkert:** Data curation, Formal analysis, Methodology, Writing – review & editing. **Esther Ruberte:** Data curation, Formal analysis, Writing – review & editing. **Michael Amann:** Methodology, Resources, Software, Writing – review & editing. **Johanna M. Lieb:** Conceptualization, Methodology, Data curation, Investigation, Writing – review & editing. **Yvonne Naegelin:** Conceptualization, Methodology, Data curation, Investigation, Writing – review & editing. **Jannis Müller:** Data curation, Formal analysis, Writing – review & editing. **Jens Kuhle:** Conceptualization, Methodology, Data curation, Investigation, Writing – review & editing. **Tobias Derfuss:** Conceptualization, Methodology, Data curation, Investigation, Writing – review & editing. **Ludwig Kappos:** Conceptualization, Methodology, Data curation, Project administration, Investigation, Writing – review & editing. **Jens Wuerfel:** Conceptualization, Data curation, Methodology, Project administration, Resources, Supervision, Writing – review & editing. **Cristina Granziera:** Conceptualization, Methodology, Project administration, Supervision, Writing – review & editing. **Özgür Yaldizli:** Conceptualization, Data curation, Formal analysis, Investigation, Methodology, Project administration, Resources, Supervision, Validation, Writing – review & editing.

## Declaration of Competing Interest

The authors declare the following financial interests/personal relationships which may be considered as potential competing interests: Tim Sinnecker is part-time employee of the Medical Image Analysis Center Basel. Sabine Schädelin has nothing to disclose. Esther Ruberte is employee of the Medical Image Analysis Center Basel. Vera Canova has nothing to disclose. Michael Amann is employee of the Medical Image Analysis Center Basel. Johanna Lieb has nothing to disclose. Yvonne Naegelin's employer, the University Hospital Basel received payments for lecturing from Celgene GmbH and Teva Pharma AG that were exclusively used for research support, not related to this study. Jannis Müller has nothing to disclose. Jens Kuhle received speaker fees, research support, travel support, and/or served on advisory boards by ECTRIMS, Swiss MS Society, Swiss National Research Foundation [320030_160221], University of Basel, Bayer, Biogen, Genzyme, Merck, Novartis, Protagen AG, Roche, Teva. Tobias Derfuss received speaker fees, research support, travel support, and/or served on Advisory Boards or Steering Committees of Novartis Pharma, Merck, Biogen, Teva, Bayer- Schering, GeNeuro, Mitsubishi Pharma, MedDay, Roche, and Genzyme; he received research support from Biogen, Novartis, Swiss National Research Foundation, University of Basel, and Swiss MS Society. Ludwig Kappos' Institution (University Hospital Basel) has received steering committee, advisory board and consultancy fees used exclusively for research support in the department, as well as support of educational activities, from Actelion, Allergan, Almirall, Baxalta, Bayer, Biogen, Celgene/Receptos, CSL-Behring, Desitin, Eisai, Excemed, F. Hoffmann-La Roche Ltd, Genzyme, Japan Tobacco, Merck, Minoryx, Novartis, Pfizer, Sanofi Aventis, Santhera and Teva; and license fees for Neurostatus-UHB products. Research at the MS Center in Basel has been supported by grants from Bayer, Biogen, the European Union, Inno-Suisse, Novartis, Roche, the Swiss MS Society and the Swiss National Research Foundation. Jens Wuerfel is CEO of the Medical Image Analysis Center Basel. Cristina Granziera has nothing to disclose. Özgür Yaldizli received grants from ECTRIMS/MAGNIMS, University of Basel, Pro Patient Stiftung University Hospital Basel, Free Academy Basel, Swiss Multiple Sclerosis Society, Swiss National Research Foundation and advisory board, lecture and consultancy fees from Roche, Sanofi Genzyme, Allmirall, Biogen and Novartis.

## Data Availability

Data will be made available on request.
