# Supplementary material for: Brain atrophy measurement over a MRI scanner change in multiple sclerosis
Source: Neuroimage Clin. 2022 Aug 10;36:103148. doi: 10.1016/j.nicl.2022.103148 (PMC9424626; doi:10.1016/j.nicl.2022.103148)
Supplement: Supplementary data 1 [file mmc1.pdf]

Supplemental material

Main manuscript: Brain atrophy measurement over a MRI scanner change in multiple sclerosis

PBVC as calculated by SIENA on lesion filled images.

Methods: T2w lesions were manually marked by trained raters in consensus reading using JIM (Xinapse Systems Ltd, West Bergholt, UK). PD/T2w images were not available in one MS patient. Lesion filling was done with “lesion_filling” as part of FMRIB Software Library (FSL, version 5.0.10, FMRIB Analysis Group, Oxford, UK) after linear registration using FLIRT (FSL, version 5.0.10, FMRIB Analysis Group, Oxford, UK, rigid registration, nearest neighbors interpolation).

```
##      nbr.val      nbr.null       nbr.na          min          max        range
##   19.0000000     0.0000000    0.0000000   -5.8342900   -3.2106750    2.6236150
##          sum        median         mean      SE.mean CI.mean.0.95          var
##  -80.5595960    -4.3039000   -4.2399787    0.1699723    0.3570986    0.5489213
##      std.dev      coef.var
##    0.7408922    -0.1747396
```

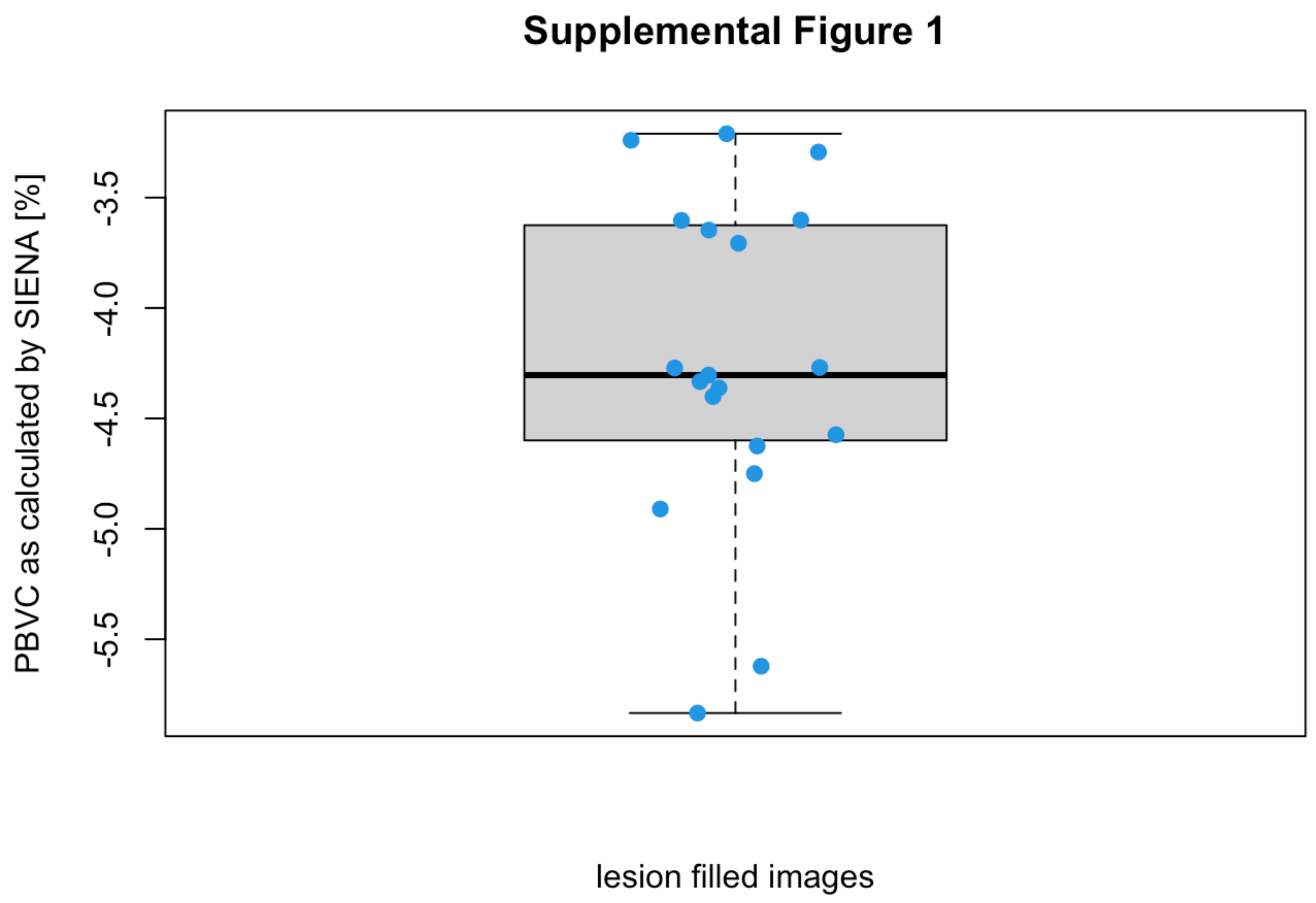

Supplemental Figure 1: Boxplot of brain volume changes on lesion filled images. The figure illustrates the distribution of PBVC as calculated by SIENA on lesion filled images. PBVC was not improved when calculated on lesion filled (median PBVC -4.2%) versus unfilled (median PBVC -4.1%) images.

PBVC as calculated by SIENA on bias field corrected and histogram matched images.

Methods: Bias field was corrected by using the N4 bias field correction algorithm, and histogram matching was done with 256 number of histogram levels 20 number of match points by using the Insight Toolkit (ITK, NumFOCUS, USA, version 5.2.1).

```
##      nbr.val      nbr.null       nbr.na          min          max        range
##   20.0000000     0.0000000    0.0000000   -8.2122100    0.6789656    8.8911756
##          sum        median         mean      SE.mean CI.mean.0.95          var
##  -51.7982429    -2.5381725   -2.5899121    0.5084552    1.0642090    5.1705339
##      std.dev      coef.var
##    2.2738808    -0.8779760
```

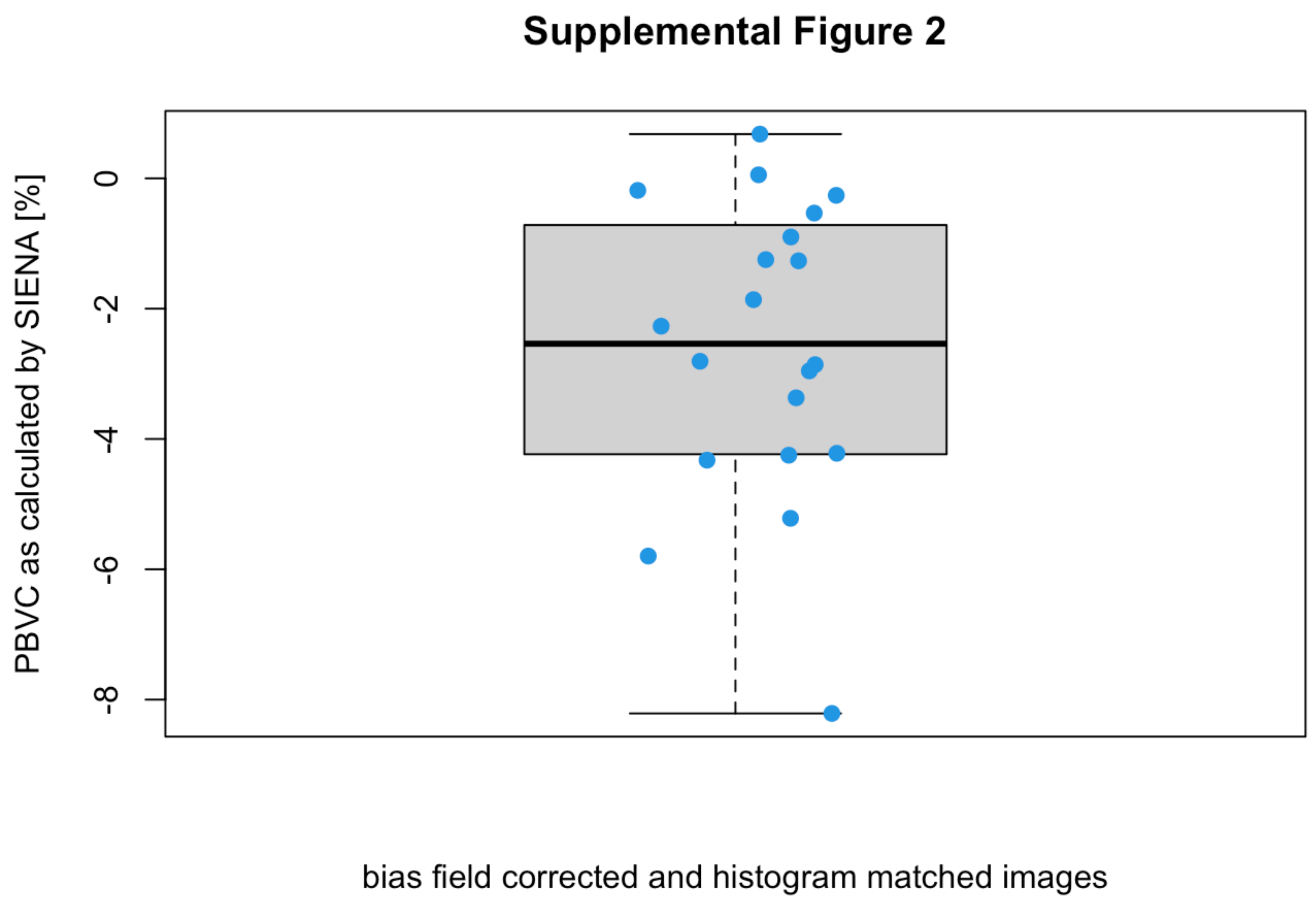

Supplemental Figure 2: Boxplot of brain volume changes on intensity adjusted images. The figure illustrates the distribution of PBVC as calculated by SIENA on intensity adjusted images. Median PBVC was lower (-2.5%) compared to raw images (-4.1%), but the method also resulted in an outlier (PBVC -8.9%).
